# Structure Related Inhibition of Enzyme Systems in Cholinesterases and BACE1 In Vitro by Naturally Occurring Naphthopyrone and Its Glycosides Isolated from *Cassia obtusifolia*

**DOI:** 10.3390/molecules23010069

**Published:** 2017-12-28

**Authors:** Srijan Shrestha, Su Hui Seong, Pradeep Paudel, Hyun Ah Jung, Jae Sue Choi

**Affiliations:** 1 Department of Food and Life Science, Pukyong National University, Busan 48513, Korea; srijanstha003@gmail.com (S.S.); seongsuhui@naver.com (S.H.S.); phr.paudel@gmail.com (P.P.); 2 Department of Food Science and Human Nutrition, Chonbuk National University, Jeonju 54896, Korea

**Keywords:** BACE1, cholinesterases, *Cassia obtusifolia*, rubrofusarin glycosides, structure-activity relationships, Alzheimer’s disease

## Abstract

*Cassia obtusifolia* Linn. have been used to improve vision, inflammatory diseases, and as hepatoprotective agents and to promote urination from ancient times. In the present study, we investigated the influence of glycosylation of components of *C. obtusifolia* and structure-activity relationships (SARs) with respect to the inhibition of acetylcholinesterase (AChE), butyrylcholinesterase (BChE), and β-site amyloid precursor protein (APP)-cleaving enzyme 1 (BACE1), which are related to Alzheimer’s disease (AD). All six *C. obtusifolia*-derived compounds, rubrofusarin (**1**), rubrofusarin 6-*O-*β-d-glucopyranoside (**2**), rubrofusarin 6-*O-*β-d-gentiobioside (**3**), nor-rubrofusarin 6-*O-*β-d-glucoside (**4**), isorubrofusarin 10-*O-*β-d-gentiobioside (**5**), and rubrofusarin 6-*O-*β-d-triglucoside (**6**) showed promising inhibitory activity against AChE/BACE1. Compounds **3** and **4** showed most significant inhibition against AChE and BACE1, respectively. The SARs results emphasized the importance of gentiobiosyl moiety in the rubrofusarin for AChE inhibition, whereas the presence of hydroxyl group at C-8 and the glucosyl moiety at the C-6 position in the nor-rubrofusarin appeared to largely determine BACE1 inhibition. Kinetics and docking studies showed the lowest binding energy and highest affinity for mixed-type inhibitors, **3** and **4**. Hydrophobic bonds interactions and the number of hydrogen bonds determined the strength of the protein-inhibitor interaction. These results suggest that *C. obtusifolia* and its constituents have therapeutic potential, and that the SARs of its active components are further explored with a view towards developing a treatment for AD.

## 1. Introduction

Alzheimer’s disease (AD) is the most predominant neurodegenerative disorder that is found in the elderly. It has been reported that more than 46.8 million people suffer from AD, and that the associated annual medical costs exceed $818 billion. In the same report, it was estimated that more than 131.5 million individuals would have AD in the year 2050 [1]. Life expectancy after clinical symptom onset is around 8.5 years [2]. But, until now, there is no effective treatment that targets the underlying molecular causes of disease. Although the absolute pathophysiological mechanism of the disease is not clear, two hypotheses have been proposed, which are, the “cholinergic hypothesis” and the “amyloid hypothesis”. According to the cholinergic hypothesis, insufficiency of cholinergic functions in the brain results in the memory impairments. The use of acetylcholinesterase (AChE) and butyrylcholinesterase (BChE) inhibitors (enzymes responsible for metabolic hydrolysis of acetylcholine), which increase the availability of acetylcholine at cholinergic synapses, offer a promising therapeutic strategy for activation of central cholinergic function [3]. On the other hand, the amyloid hypothesis states that amyloid-β peptide (Aβ) accumulation in the brain is responsible for the pathogenesis of AD. Amyloid plaques, which is primarily composed of Aβ, gradually form in the brains of AD patients, and mutations in amyloid precursor protein, presenilin 1 and 2 cause early-onset familial AD by directly increasing toxic, plague-promoting Aβ production. β-Site amyloid precursor protein-cleaving enzyme 1 (BACE1) displays all of the functional properties of β-secretase, and is a key enzyme in the Aβ formation, and thus, BACE1 is an appropriate target site for drug [4].

Cassia semen (sicklepod) is the seed of an annual plant *Cassia obtusifolia* L. (Leguminosae), which is grown widely in Korea and China. Traditionally, Cassia seed has been used to treat dizziness, headache, improve vision, and as liver-calming [5], and has been reported to have antioxidant effects [6], neuroprotection effects in Parkinson’s disease models [7], to attenuate scopolamine induced memory impairment or transient cerebral hypoperfusion in mice [8], ameliorate amyloid β-induced synaptic dysfunction through anti-inflammatory and Akt/GSK-3β pathways [9], to attenuate secondary calcium dysregulation and to have neuroprotective effect against mitochondrial toxin (which has been implicated in pathogenesis of AD) in mouse primary hippocampal cultures [10], to have antimicrobial activity [11], and to inhibit protein glycation and aldose reductase activity in vitro [12].

Rubrofusarin and its derivatives that were isolated from *C. obtusifolia* have been reported to have anti-cancer effects [13], hepatoprotective effects [14], advanced glycation end products inhibitory [15], 1,1-diphenyl-2-picrylhydrazyl radical scavenging [16] antioxidant [17], anti-AD [18], anti-diabetes [19], anti-estrogenic [20], and antimycobacterial [21] activities. Many investigations have been performed on the SARs of flavonoids, but nothing has yet been published on the SARs of naphthopyrone and its derivatives (rubrofusarin being categorized in the naphthopyrone class). Previously, we reported the effects of the glycosylation of naringenin on the inhibition of enzyme systems that are related to diabetes (protein tyrosine phosphatase 1B and α-glucosidase) and on glucose uptake in the insulin-resistant state [22]. Further, we reported on the inhibitory activities of major chemical constituents that are isolated from *C. obtusifolia* against BACE1 and cholinesterase [18]. Among the various fractions, *n*-butanol (BuOH) fraction exhibited the most potent inhibitory activities against BACE1 and cholinesterase. Different compounds were isolated from this fraction and among them, toralactone gentiobioside showed moderate activity, whereas nor-rubrofusarin 6-*O-*β-d-glucoside (**4**) exhibited most potent BACE1 inhibitory activity. It is interesting to note that both compounds share structural similarity, but exhibit different activity, and there were reports regarding the presence of more structural analogs in the cassia seeds [18].

**Figure 1:**
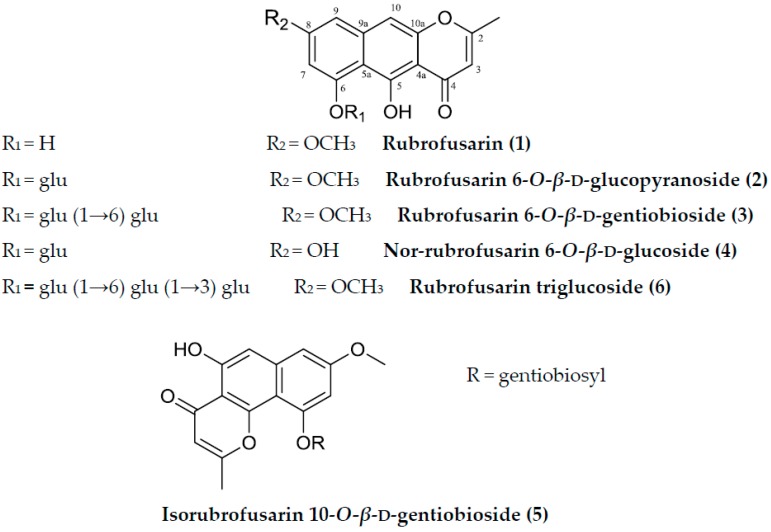
Structures of the compounds isolated from *C. obtusifolia*.

**Figure 2:**
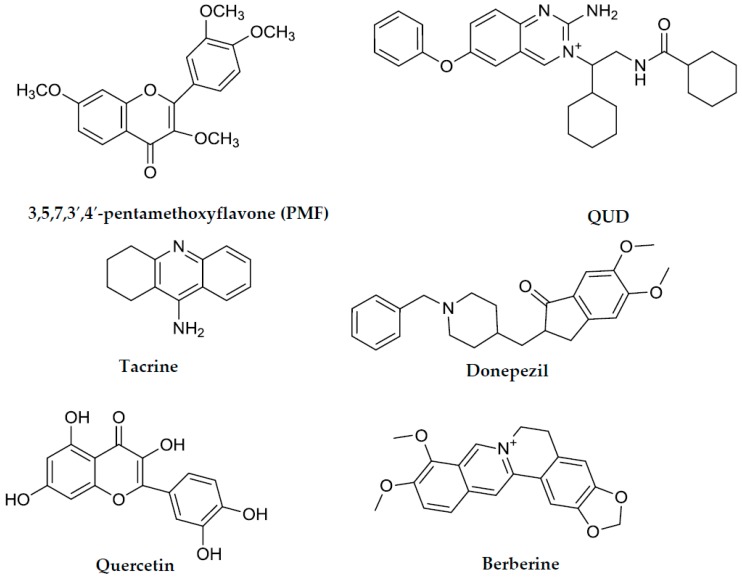
Structures of the positive controls used in in vitro assays and in silico molecular docking analysis.

Structure-activity relationships (SARs) link molecular structures with properties, effects, or biological activities [23]. SARs studies are fundamental to many phases of drug discovery from primary screening to lead development. These studies start with the identification of a collection of molecules of interest, and then following this with studies that establish relationships between molecular structures and observed activities to identify structural features that enhance properties or functionalities of interest [24]. Due to time and cost restraints, it is not possible to test all of the compounds of interest thoroughly, and thus, SAR studies help predict biological responses and to determine the direction of future developments in a cost and time effective manner [25]. The biological activities of a new chemical species can often be predicted from its molecular structure using other similar compound’s database. Accordingly, SAR studies are powerful tool for understanding functional implications of unknowns when molecular similarities are evident. Therefore, we performed further analysis to isolate more similar compounds (naphthopyrones) accordingly, with the aim of investigating the SARs of naphthopyrones isolated from *C. obtusifolia* (Figure 1 and Figure 2). In the present study, we selected rubrofusarin and its derivatives isolated from *C. obtusifolia* in order to investigate the influences of: (i) glycosylation; (ii) C-8 methoxyl group; and, (iii) pyrone ring arrangement at the naphthalene ring on their biological activities against cholinesterases and BACE1. In addition, we performed kinetic and molecular studies to validate the mechanism of the interaction between compounds and the active site of enzymes.

## 2. Results

### 2.1. Inhibitory Activities of Rubrofusarin and Its Derivatives against AChE, BChE, and BACE1

In order to evaluate the anti-AD potential of the isolated rubrofusarin and its five derivatives, we evaluated their abilities to inhibit AChE, BChE, and BACE1. Results are summarized in Table 1. Of the derivatives, **3** showed the most potent activity against AChE with an IC_50_ of 15.94 ± 0.32 µM whereas **2** had least inhibitory activity (148.08 ± 2.09 µM). **4**–**6** exhibited moderate inhibition with IC_50_ values of 86.05 ± 2.01, 83.52 ± 1.56, and 82.31 ± 1.63 µM, respectively, when compared with the positive control berberine (0.68 ± 0.01 µM). Next, we investigated the BChE inhibitory activities of rubrofusarin and its derivatives. Unfortunately, only **3** inhibited BChE with an IC_50_ value of 141.15 ± 1.23 µM. Finally, the isolated compounds were investigated for their activity against BACE1. Interestingly, **4** (methoxyl group substituted by hydroxyl at C-8 position) was most potent, with an IC_50_ value of 14.41 ± 2.87 µM (the positive control quercetin had an IC_50_ value of 21.42 ± 1.04). **1** and **3** demonstrated moderate inhibition with IC_50_ values of 90.01 ± 2.38 and 85.66 ± 3.98 µM, respectively. However, **5** (an isomer of **3**) and **6** demonstrated no significant inhibitory activity at concentrations up to 200 µM.

### 2.2. Enzyme Kinetic Analysis with AChE and BACE1

**Figure 3:**
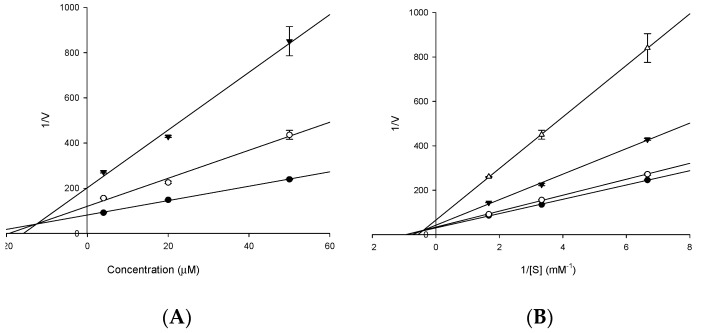
Dixon plots and Lineweaver-Burk plots for acetylcholinesterase (AChE) inhibition by **3**. The results showed the effects of presence of different concentrations of the substrate (0.6 (●), 0.3 (○), and 0.1 mM (▼)) for (**A**) and the effect of presence of different concentration of **3** (0 (●), 4 (○), 20 (▼), and 50 µM (△)) for (**B**).

**Figure 4:**
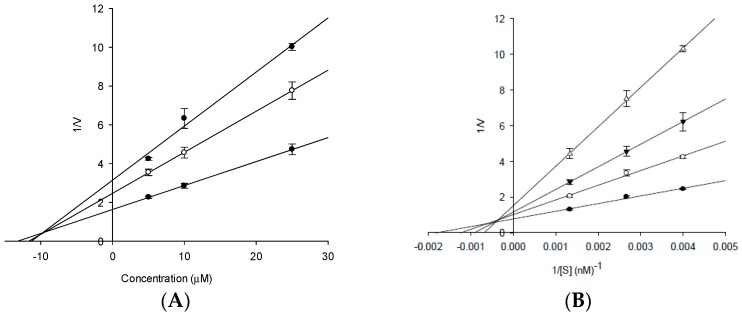
Dixon plots and Lineweaver-Burk plots for β-site amyloid precursor protein-cleaving enzyme 1 (BACE1) inhibition by **4**. The results showed the effects of presence of different concentrations of the substrate (252 (●), 375 (○), and 750 nM (▼)) for (**A**) and the effect of presence of different concentration of **4** (0 (●), 5 (○), 10 (▼), and 25 µM (△)) for (**B**).

Since compound **3** showed high AChE inhibitory activity, it was subjected to an enzyme kinetic study. According to the Lineweaver-Burk plot (Figure 3), it exhibited mixed type inhibition against AChE. Moreover, the Dixon plot revealed a *K_i_* value of 12.83 µM. Since **4** showed significantly high inhibitory activity against BACE1, it was also subjected to enzyme kinetic studies (Figure 4). Its Dixon plot revealed a *K_i_* value of 10.01 µM. The results of the enzyme kinetic analysis of **3** and **4** against AChE and BACE1 are shown in Table 1. In general, compounds with lower *K_i_* value are preferred and are more active inhibitors against AChE and BACE1.

### 2.3. Molecular Docking Simulation in AChE inhibition

**Figure 5:**
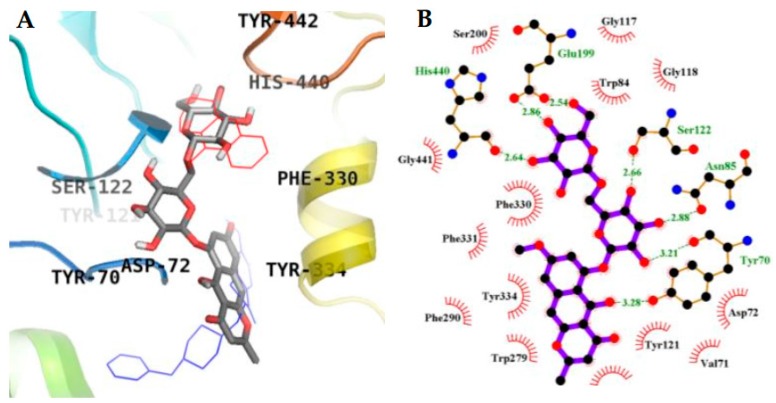
Inhibition mode of **3** (**A**) for the AChE active site with tacrine (red line) and donepezil (blue line) (**A**); 2D ligand interaction diagram of AChE mixed inhibition by **3** (**B**). Dashed green lines indicate H-bonds. Carbons are in black, nitrogens in blue, and oxygens in red.

**Figure 6:**
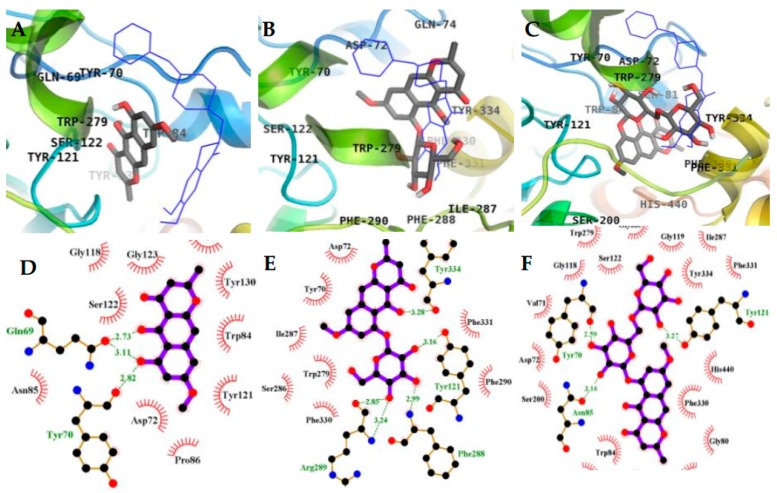
Inhibition mode of **1** (**A**); **2** (**B**); and **3** (**C**) for the AChE allosteric site with donepezil (blue line) (**A**). 2D ligand interaction diagram of AChE allosteric inhibition by **1** (**D**); **2** (**E**); and **3** (**F**). Dashed green lines indicate H-bonds. Carbons are in black, nitrogens in blue, and oxygens in red.

Molecular docking simulation provides a means of understanding the protein–ligand interaction geometrics at a molecular level. Tacrine and donepezil were used as standard ligands to validate the AutoDock 4.2 results. Both are potent reversible inhibitors of AChE with IC_50_ values of 0.25 µM (tacrine) [27] and 0.005 µM (donepezil) [28], as mentioned in Table 1. The binding energy of **1**–**3** with interacting residues are described in Figure 5 and Figure 6 and Table 2. As shown in Table 2, the top binding energy of AChE-**3** complex had a −9.57 Kcal/mol in the allosteric inhibition mode. Three hydrogen bonds were observed between the oxygen group of TYR70, ASN85, and TYR121, and the hydroxyl group of **3** with the bond distance of 2.59, 3.16, and 3.27 Å, respectively. Especially, TYR70 is important peripheral anionic site (PAS) residue of AChE. In addition, **3** and TRP279 residue (included in PAS) of AChE participated in hydrophobic interactions (Figure 6C,F). The second highest binding mode of AChE-**3** complex had a −9.06 Kcal/mol binding energy having seven H-bonds with interacting residues of TYR70 (PAS residue), ASN85, SER122, GLU199, and HIS440 (residue of catalytic anionic site) in case of mixed-mode inhibition (Figure 5A,B). In addition, hydrophobic interactions were also observed between VAL71, ASP72, GLN74, TRP84, GLY117, GLY118, TYR121, SER200, PHE290, PHE330, PHE331, TYR334, and GLY441 residues and **3**. As shown in Figure 5B, several H-bonds were formed between enzyme and gentiobiosyl moiety of **3**. AutoDock 4.2 simulations results from interactions between the **1** and **2** with AChE are illustrated in Figure 6. The results demonstrated that AChE-**1** complex at the allosteric site has three H-bonding interactions between GLN69 and TYR70 (PAS residue) of AChE and **1** (binding energy: −7.95 Kcal/mol). Furthermore, ASP72, TRP84, ASN85, PRO86, GLY117, GLY118, TYR121, SER122, GLY123, LEU127, and TYR130 of AChE were participated in hydrophobic interactions (Figure 6A,D). Similarly, the AChE-**2** complex had a binding energy of −7.51 Kcal/mol and five H-bonds due to interactions with residues TYR121, TYR334, PHE288, and ARG289. In addition, **2** showed hydrophobic interactions, with important PAS residues including TYR70, ASP72, and TRP279 (Figure 6B,E). These results indicated that **1** and **2** are allosteric inhibitors, and **3** is mixed type inhibitor.

### 2.4. Molecular Docking Simulation in BACE1 Inhibition

**Figure 7:**
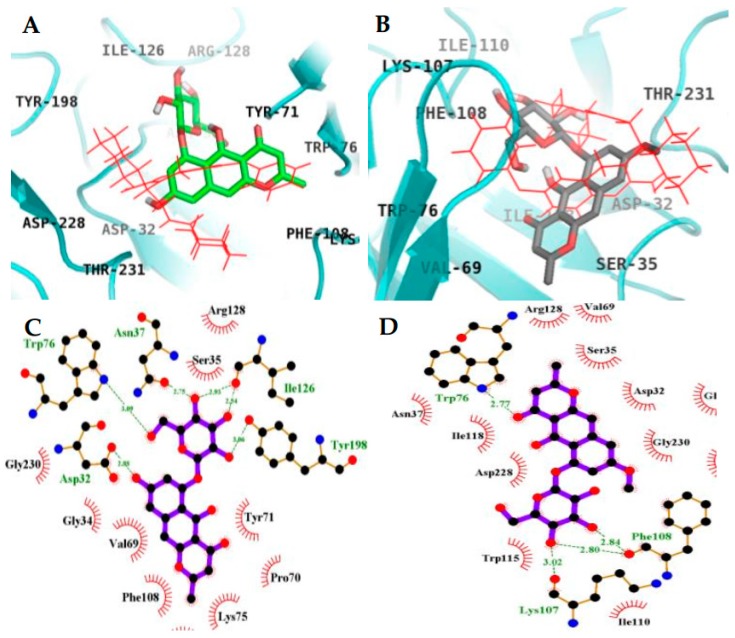
Inhibition mode of **4** (**A**) and **2** (**B**) for the BACE1 catalytic site with 2-amino-3-{(1*R*)-1-cyclohexyl-2-[(cyclohexylcarbonyl) amino]ethyl}-6-phenoxyquinazolin-3-ium (QUD) (red line) (**A**). 2D ligand interaction diagrams of BACE1 catalytic inhibition by **4** (**C**) and **2** (**D**). Dashed green lines indicate H-bonds. Carbons are in black, nitrogens in blue, and oxygens in red.

**Figure 8:**
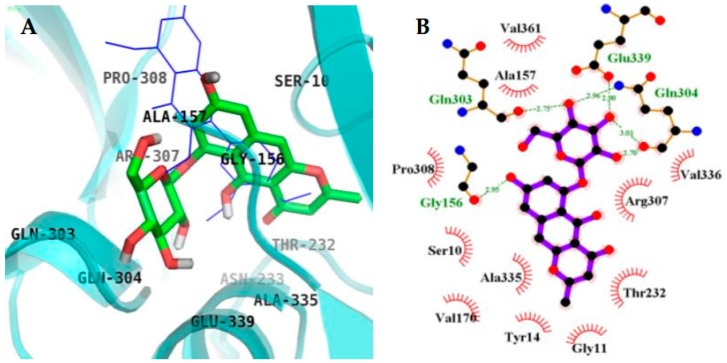
Inhibition mode of **4** for the BACE1 allosteric site with PMF (blue line) (**A**). 2D ligand interaction diagram of BACE1 allosteric (**B**) inhibition by **4**. Dashed green lines indicate H-bonds. Carbons are in black, nitrogens in blue, and oxygens in red.

The binding energy of **4** and **2** with the interacting residues, including H-bond interacting residues and van der Waals interactions, and the numbers of H-bonds are listed in Figure 7 and Figure 8, and Table 3. 2-Amino-3-{(1*R*)-1-cyclohexyl-2-[(cyclohexylcarbonyl)amino]ethyl}-6-phenoxyquinazolin-3-ium (QUD) (catalytic inhibitor) and 3,5,7,3′,4′-pentamethoxyflavone (PMF) (allosteric inhibitor) were used as positive ligands. QUD is the selective non-peptic BACE1 ligand reported till date according to the RCSB Protein Data Bank, and PMF is reported to be a noncompetitive inhibitor (IC_50_ = 59.8 µM) [30]. According to Autodock 4.2 simulation results, the top binding pose of BACE1-**4** complex had a −8.34 Kcal/mol in the allosteric inhibition mode and formed six H-bonds with GLN303, GLN304, GLU339, and GLY156 (Figure 8). SER10, ALA335, VAL170, TYR14, GLY13, GLY11, THR232, ARG307, VAL336, VAL361, ALA157, and PRO308 residues were found to participate in hydrophobic interactions. H-bond interaction between hydroxyl group of C-5 of **4** and GLY156 residue seem to play a crucial role in the binding affinity of **4**. In addition, catalytic inhibition mode of **4** also observed with a −6.61 Kcal/mol of binding energy. This complex formed six H-bonds with ASP32, TRP76, ASN37, ILE126, and TYR198 residues of BACE1 (Figure 7C). Especially, important catalytic residue ASP32 and hydroxyl group of C-5 of **4** interacted via H-bonds, whereas methoxyl grouop of C-5 of **2** did not interact with ASP32. This difference could greatly affect the inhibitory activity and the binding affinity between **4** and **2** against BACE1.

## 3. Discussion

Considerable research efforts have been directed towards discovering the cause of AD in recent years, with the ultimate hope of developing safe and effective pharmacological treatments. Symptom control, maintaining functional status, improving quality of life, minimizing the adverse drug effect, and reducing caregiver stress are the main target in the management of AD. ACh, a neurotransmitter plays an essential role in memory and learning, and AChE is the enzyme that hydrolyzes ACh at the cholinergic synapses in the central and peripheral nervous system. AChE inhibitors promote an increase in the concentration and the duration of action of synaptic ACh and thus, enhance cholinergic transmission through the activations of synaptic nicotinic and muscarinic receptors. Reports suggest that AChE plays a key role during the early development of senile plague, as was revealed by the finding that AChE accelerates β-amyloid peptide (Aβ) deposition [31]. Second generation ChEs inhibitors, like donepezil, rivastigmine, metrifonate, and galantamine are now used for the symptomatic relief of AD, and provide benefits as those reported for tacrine, but with more favorable clinical profiles [3]. The AChE inhibitors donepezil and rivastigmine have a similar mode of action (both increase the concentration of Ach at the neurotransmitter sites). Galantamine also acts in the same manner but also modulates activity at the nicotinic receptor [32]. Nevertheless, available drugs have side effects, like gastro-intestinal disturbances, nausea, vomiting, and diarrhea, and also have bioavailability issues [33]. Nowadays, natural medicinal drugs are reclaiming their position as the primary source of treatment as alternatives to synthetic agents [34,35,36,37]. Research on *C. obtusifolia* indicates that it has beneficial effects in AD, diabetes, and Parkinson’s disease.

The sugar moiety attached to a specific position on the aglycone core plays an important role in the structural biodiversity of natural products. These sugar components usually participate in the molecular recognition of its cellular target, and thus, they are important for biological activity. These sugars can be linked to an aglycone as monosaccharides, disaccharides or oligosaccharides of variable sugar lengths via C-, N- or *O*-glycosylation (*O*-glycosylation is the most common) [38]. In addition, several reports have been issued on the influence of methoxyl group on biological activity [39,40,41]. 

Previously, we reported the anti-AD and anti-diabetic activities of major constituents from *C. obtusifolia* [18,19]. In the present study, we investigated the anti-AD activity of rubrofusarin and its derivatives isolated from *C. obtusifolia* by performing ChEs and BACE1 inhibitory assay (Table 1). All six of the tested compounds showed promising inhibitory activity against AChE with IC_50_ ranging from 15.94 to 148.08 µM. The abilities of selected compounds to inhibit AChE followed in decreasing order, **3** > **6** > **5** > **4** > **1** > **2**, respectively. Interestingly, **3,** which contains two sugar moieties, inhibited AChE nine times more effectively than **2** (having one sugar moiety), eight times more than **1** (having no sugar moiety), and five times than **5** (an isomer of **3**), **6**, and **4**, respectively. Examination of the SAR of rubrofusarin and its derivatives isolated from *C. obtusifolia* indicated that introduction of gentiobiosyl moiety in the **1** scaffold increases AChE inhibitory activity, whereas further addition of glucose in **3** decreases AChE inhibitory activity, indicating the importance of gentiobiosyl moiety and the length of sugar moeity at C-6. This results shows that gentibiosyl moiety may be the optimum sugar length for the particular biological activity. By comparing AChE inhibitory activity of **3** with its isomer **5** (IC_50_ = 83.52 µM), which differs with respect to the arrangement of pyrone ring in the naphthalene group (i.e., the pyrone ring attached at β-position at naphthalene ring in **5** and at γ-position in **3**) revealed that the arrangement of pyrone at naphthalene ring and sugar position greatly influence activity. In addition, the introduction of the hydroxyl group at C-8 position of **2** (IC_50_ = 148.08 µM) in **4** (IC_50_ = 86.05 µM) increases the inhibitory activity, indicating the importance of the hydroxyl group at C-8 position for its activity. Similar results regarding the correlation between methoxyl group at C-6/C-7/C-8 position and AChE inhibitory activity was also obtained in our previous study [42]. Luo et al. also found a relation between the presence of C-1 methoxyl group in the new series of arylnaphthalenes with a decrease in antitumor activity [43].

In order to support SARs of compounds obtained from experimental data and to evaluate the binding site-directed inhibition of AChE, kinetic and docking studies were performed. Molecular docking simulation is a potent and progressively important tool for drug discovery [44,45] and can be used for screening and identification of probable biological active chemical moiety from natural product databases [46]. We obtained docking score using Autodock 4.2 to estimate the strength of the different protein-ligand complex interaction. For the docking studies, we used tacrine and donepezil as respective catalytic and allosteric inhibitor, to validate the results for AChE. These studies reveal that **3** exhibited a mixed type of inhibition, whereas **1** and **2** exhibited allosteric type inhibition. Molecular docking of **3** produced an interesting result. Generally, mixed type of inhibition implies binding with the catalytic and allosteric site. However, docking at the catalytic site revealed the presence of both allosteric and catalytic interacting residues, suggesting the compound’s preference for the allosteric site over catalytic site. In addition, docking results showed a lower binding energy for **3** (mixed type inhibition (−9.06 Kcal/mol); allosteric inhibition mode (−9.57 Kcal/mol)) than **2** (−7.51 Kcal/mol) and **1** (−7.95 Kcal/mol) for allosteric inhibition, which supported a greater affinity of compound **3** and its greater binding capacity to the allosteric site. Further, **3** bound to allosteric site had a similar number of H-bonds as **1** and **2** (Table 2, Figure 5 and Figure 6). However, van der Waals interactions between **3** and interacting residues were almost twice that of the other derivatives, indicating the importance of van der Waals interactions in addition to H-bond in terms of strengthening the protein-ligand complex, and suggesting that this might be the reason for its better activity than other derivatives for AChE. Previously, we reported that the activity of isovitexin is greater than its aglycone apigenin in terms of diabetic complication and anti-AD activity [47]. Also, compound **3** was found to have twice the radical scavenging activity of 4 [16].

In addition, rubrofusarin and its derivatives were tested for their BACE1 inhibitory effects. All of the compounds, except **5**, showed promising inhibitory activity, with IC_50_ values ranging from 14.41 to 190.63 µM. However, compound activities did not follow the pattern observed for AChE. Instead, activities followed the order **4** > **3** > **1** > **2** > **6** > **5**. **4** showed most potent inhibitory activity against BACE1 (IC_50_ = 14.41 µM), which was more potent than of quercetin (21.42 µM; positive control). Analysis of SARs indicated that the introduction of a methoxyl group at C-8 position of **4** (IC_50_ = 86.05 µM) in **2** (IC_50_ = 148.08 µM) greatly decrease inhibitory activity, signifying the presence of hydroxyl group at C-8 position, like that observed in AChE activity, was essential for BACE1 inhibitory activity. In addition, when comparing **5** (>200 µM) and **3** (85.66 µM) against BACE1, revealed that the normal orientation (pyrone ring at γ-position of naphthalene ring) is essential for the BACE1 inhibitory activity, whereas β-position attachment of pyrone ring resulted in a decrease or loss in activity.

Further, kinetic and docking studies of **4** showed mixed type of inhibition, whereas **2** demonstrated catalytic inhibition. QUD and PMF were used to simulate catalytic and allosteric site, respectively, as they are the reported selective inhibitors of BACE1. BACE1 has two main domains, that is, *N*-terminal domain and *C*-terminal domain with numerous detailed sub-regions distributed between them [48]. The two aspartate residues, ASP32 and ASP228, in the cleft of the active site conserve the catalytic site of BACE1 [49] whereas SER10, THR 232, VAL336, and ALA157 compose the allosteric site of BACE1 [30]. The BACE1-**4** complex had a calculated binding energy −6.61 Kcal/mol attributed to six hydrogen bonds in the catalytic site, whereas in the allosteric site, six hydrogen bonds were formed with −8.34 Kcal/mol binding energy (Table 3). In contrast, **2** demonstrated competitive inhibition (binds to catalytic site of enzyme) and formed four H-bonds (lesser) and binding energy of −5.38 Kcal/mol (higher) when compared to that of **4**. This implies the prominence of hydrophobic bonds, hydrogen bond distance, and binding energy, in addition to H-bond, underlies the strength of the protein-ligand interaction and the positioning of the inhibitor in the specific pocket.

## 4. Materials and Methods 

### 4.1. General Experimental Procedures

The ^1^H- and ^13^C-NMR spectra were recorded in methanol-*d*_4_ (CD_3_OD-*d*_4_) and dimethyl sulfoxide (DMSO-*d*_6_) on a JEOL JNM ECP-400 spectrometer (Jeol, Tokyo, Japan) at 400 MHz and 100 MHz, respectively. Column chromatography was performed using Diaion HP-20, Sephadex LH-20 (20–100 µm) (Sigma, St. Louis, MO, USA), 70–230 mesh silica (Si) gel 60 (Merck, Darmstadt, Germany), and LiChroprep RP-18 (40–63 µm, particle size) (Merck, Darmstadt, Germany). All of the TLC were performed on a precoated Merck Kiesel gel 60 F_254_ plates (20 × 20 cm, 0.25 mm) and RP-18 F_254S_ plates (5 × 10 cm) (Merck, Darmstadt, Germany). The spray reagent was 25% H_2_SO_4_.

### 4.2. Chemicals and Reagents

Electric-eel AChE (EC 3.1.1.7), horse-serum BChE (EC 3.1.1.8), acetylthiocholine iodide (AChI), butyrylthiocholine chloride (BCh), 5,5′-dithiobis-(2-nitrobenzoic acid) (DTNB), quercetin, and berberine were purchased from Sigma Co. (St. Louis, MO, USA). A BACE1 fluorescence resonance energy transfer (FRET) assay kit (β-secretase) was purchased from Pan Vera Co. (Madison, WI, USA). All of the chemicals and solvents used in column chromatography and assays were of reagent grade and were purchased from commercial sources.

### 4.3. Plant Material

Raw seeds of *Cassia obtusifolia* were purchased (Omni Herb Co., Daegu, Korea) and identified by Prof. J. H. Lee (Dongguk University, Gyeongju, Korea). A voucher specimen (No. 20160302) was registered and deposited in the Prof. J. S. Choi laboratory (Pukyong National University, Busan, Korea).

### 4.4. Extraction, Fractionation, and Isolation

The seeds of *C. obtusifolia* (1.8 kg) were refluxed in methanol (MeOH) (3 h × 5 times). The total filtrate was then dried using evaporator under reduced pressure to produce the MeOH extract. The extract was suspended in distilled water and was continuously partitioned with dichloromethane to get the dichloromethane fraction and water residue. The water residue (119.02 g) was subjected to column chromatography (Diaion HP-20) and eluted with distilled water, 40% MeOH, 60% MeOH and 100% MeOH, gradually to obtain four subfractions of 56.01 g, 30.19 g, 22.42 g, and 2.72 g, respectively. The 40% MeOH subfraction was subjected to Si gel chromatography and eluted with ethyl acetate:MeOH:water (EMW = 600:99:81) to yield 20 subfractions. Subfraction 4 was subjected to Si gel chromatography, using dichloromethane:MeOH:water (CMW = 7:1:0.1) to obtain **4** (12 mg). Subfraction 5 was subjected to repeated Si gel chromatography using EMW and CMW in various ratio to get **2** (10 mg). Subfraction 9 was subjected to repeated chromatography using EMW and CMW in various ratio to get **5** (30 mg). Subfraction 10 was chromatographed over Si gel using EMW (600:99:81) to get **3** (54 mg). The precipitate that was obtained from subfraction 14 was washed with MeOH continuously to get **6** (50 mg). The 100% MeOH subfraction was subjected to Si gel chromatography and eluted with dichloromethane: MeOH (CM = 30:1) to yield 10 subfractions. Subfraction 1 was subjected to Si gel chromatography, and eluted with CMW (15:1:0.1) to get **1** (35 mg). All of the compounds were characterized and identified by spectroscopic methods (^1^H- and ^13^C-NMR) and also matched with the previously published data [15,50,51]. The structures of these compounds are shown in Figure 1.

*Compound* **1**: Orange needles; ^1^H-NMR (400 MHz, CD_3_OD) δ: 6.98 (1H, s, H-2), 6.88 (1H, d, *J* = 1.95 Hz, H-8), 6.69 (1H, d, *J* = 1.95 Hz, H-9), 6.04 (1H, s, H-6), 2.38 (3H, s, CH_3_), 3.99 (3H, s, OCH_3_). ^13^C-NMR (100 MHz, CD_3_OD) δ: 170.12 (C-2), 106.7 (C-3), 184.2 (C-4), 103.3 (C-4a), 163.1 (C-5), 107.4 (C-5a), 159.5 (C-6), 101.1 (C-7), 163.3 (C-8), 98.5 (C-9), 141.0 (C-9a), 101.4 (C-10), 153.0 (C-10a), 20.4 (CH_3_) and 55.4 (OCH_3_).

*Compound* **2**: Yellow needles; ^1^H-NMR (400 MHz, CD_3_OD) δ: 6.86 (lH, s, H-10), 6.80 (1H, d, *J* = 2.1 Hz, H-9), 6.75 (1H, d, *J* = 2.1 Hz, H-7), 6.34 (1H, s, H-3), 5.20 (lH, d, *J* = 7.6 Hz, glucosyl H-1′), 3.90 (3H, s, OCH_3_) and 2.56 (3H, s, CH_3_). ^13^C-NMR (100 MHz, CD_3_OD) δ: 170.0 (C-2), 110.6 (C-3), 184.5 (C-4), 101.6 (C-4a), 163.0 (C-5), 106.5 (C-5a), 157.7 (C-6), 100.7 (C-7), 163.0 (C-8), 109.7 (C-9), 142.4 (C-9a), 102.0 (C-10), 152.3 (C-10a), 102.0 (C-1′), 75.1 (C-2′), 78.4 (C-3′), 71.2 (C-4′), 78.3 (C-5′), 62.4 (C-6′), 56.0 (OCH_3_), 20.4 (CH_3_).

*Compound* **3**: Yellow needles; ^1^H-NMR (400 MHz, DMSO-*d*_6_) δ: 2.39 (3H, s, CH_3_), 3.87 (3H, s, OCH_3_), 5.07 (1H, d, *J* = 7.7 Hz, glucosyl H-1′), 4.20 (1H, d, *J* = 8.1 Hz glucosyl H-1″), 6.80 (1H, d, *J* = 2.2 Hz, H-7), 6.93 (1H, d, *J* = 2.2 Hz, H-9). ^13^C-NMR (100 MHz, DMSO-*d*_6_) δ: 168.8 (C-2), 106.7 (C-3), 183.7 (C-4), 101.1 (C-4a), 161.9 (C-5), 107.7 (C-5a), 157.6 (C-6), 100.8 (C-7), 161.1 (C-8), 99.7 (C-9), 140.3 (C-9a), 103.6 (C-10), 152.4 (C-10a), 20.2 (CH_3_), 55.5 (OCH_3_), 103.6 (C-1′), 73.5 (C-2′), 76.9 (C-3′), 70.1 (C-4′), 75.5 (C-5′), 68.6 (C-6′), 99.7 (C-1″), 73.5 (C-2″), 76.4 (C-3″), 69.6 (C-4″), 76.7 (C-5″), 61.0 (C-6″).

*Compound* **4**: Yellow powder; ^1^H-NMR (400 MHz, DMSO-*d*_6_) δ: 2.36 (3H, s, CH_3_), 6.12 (1H, s, H-3), 6.72 (1H, d, *J* = 2.0 Hz, H-9), 6.68 (1H, d, *J* = 2.1 Hz, H-7), 7.06 (1H, s, H-10), 5.07, (1H, d, *J* = 7.8 Hz, glucosyl H-1′). ^13^C-NMR (100 MHz, DMSO-*d*_6_) δ: 168.6 (C-2), 106.5 (C-3), 183.7 (C-4), 103.0 (C-4a), 162.1 (C-5), 106.9 (C-5a), 158.3 (C-6), 101.3 (C-7), 159.7 (C-8), 102.5 (C-9), 140.5 (C-9a), 100.0 (C-10), 152.3 (C-10a), 20.1 (CH_3_), 101.2 (C-1′), 73.5 (C-2′), 76.4 (C-3′), 69.6 (C-4′), 77.3 (C-5′), 60.7 (C-6′).

*Compound* **5**: Yellow powder; ^1^H-NMR (400 MHz, DMSO-*d*_6_) δ: 6.47 (1H, s, H-3), 6.93 (1H, s, H-6), 6.90 (1H, d, *J* = 2.1 Hz, H-7), 6.80 (1H, d, *J* = 2.1 Hz, H-9), 5.15 (1H, d, *J* = 7.5 Hz, glucosyl H-1′), 4.19 (1H, d, *J* = 7.6 Hz, glucosyl H-1′′), 3.87 (3H, s, OCH_3_), 2.53 (3H, s, CH_3_). ^13^C-NMR (100 MHz, DMSO-*d*_6_) δ: 168.35 (C-2), 109.62 (C-3), 182.30 (C-4), 155.59 (C-5), 104.89 (C-6), 99.52 (C-7), 161.10 (C-8), 100.04 (C-9), 156.05 (C-10), 155.15 (C-11), 108.08 (C-12), 140.26 (C-13), 104.89 (C-14), 55.47 (COCH_3_), 19.81 (CH_3_), 99.52 (C-1′), 73.52 (C-2′), 76.89 (C-3′), 70.08 (C-4′), 75.42 (C-5′), 68.69 (C-6′), 103.53 (C-1′′), 73.64 (C-2′′), 76.65 (C-3′′), 69.53 (C-4′′), 76.77 (C-5′′), 61.04 (C-6′′).

*Compound* **6**: Yellow powder; ^1^H-NMR (400 MHz, DMSO-*d*_6_) δ: 6.20 (1H, s, H-3), 7.19 (1H, s, H-10), 5.01 (1H, d, *J* = 7.5 Hz, glucosyl H-1′), 4.32 (1H, d, *J* = 7.5 Hz, glucosyl H-1′′), 4.30 (1H, d, *J* = 7.5 Hz, glucosyl H-1′′′), 6.94 ( 1H, d, *J* = 2.2 Hz, H-9), 6.79 (1H, d, *J* = 2.2 Hz, H-7), 2.39 (3H, s, CH_3_), 3.87 (3H, s, OCH_3_). ^13^C-NMR (100 MHz, DMSO-*d*_6_) δ: 168.89 (C-2), 106.7 (C-3), 183.8 (C-4), 103.6 (C-4a), 161.9 (C-5), 107.7 (C-5a), 157.6 (C-6), 101.2 (C-7), 161.07 (C-8), 100.8 (C-9), 140.3 (C-9a), 101.2 (C-10), 152.4 (C-10a), 20.2 (CH_3_), 55.5 (OCH_3_), 101.2 (C-1′), 73.4 (C-2′), 76.3 (C-3′), 69.6 (C-4′), 75.6 (C-5′), 68.5 (C-6′), 102.6 (C-1′′), 72.2 (C-2′′), 88.1 (C-3′′), 68.3 (C-4′′), 76.0 (C-5′′), 60.6 (C-6′′), 104.1 (C-1′′′), 73.8 (C-2′′′), 76.9 (C-3′′′), 70.1 (C-4′′′), 76.3 (C-5′′′), 61.0 (C-6′′′).

### 4.5. In Vitro Ches Enzyme Assay

The inhibitory activities of isolated compounds against ChEs were measured using the spectrophotometric method, as described previously [52]. Briefly, AChI and BCh were used as the substrates to assay the inhibition of AChE and BChE, respectively. Each reaction mixture contained 140 µL of sodium phosphate buffer (pH 8.0); 20 µL of compounds (final conc., 100 µM); and, 20 µL of either AChE or BChE solution, which was mixed and incubated for 15 min at room temperature. Compounds and the positive control (berberine) were dissolved in 10% analytical grade DMSO. Reactions were initiated by the addition of 10 µL of DTNB and 10 µL of either AChI or BCh. The hydrolysis of AChI or BCh was monitored by following the formation of the yellow 5-thio-2-nitrobenzoate anion at 412 nm for 15 min, which was generated via reaction of DTNB with thiocholine and was released from the respective enzymatic hydrolysis by either AChI or BCh. All of the reactions were performed in triplicate and recorded in 96-well microplate format using a microplate spectrophotometer (Molecular Devices, Sunnyvale, CA, USA). Percent inhibition was calculated as (1-S/E) × 100, where E and S were the respective enzyme activities without and with the tested sample, respectively. ChE inhibitory activities were expressed in terms of the IC_50_ value (µM required to inhibit hydrolysis of the substrate, AChI or BCh, by 50%), as calculated from the log-dose inhibition curve.

### 4.6. In Vitro BACE1 Enzyme Assay

Assays were performed according to the supplied manual (Pan Vera Co.) with selected modifications, as described previously [53]. Briefly, mixtures of 10 µL of assay buffer (50 mM sodium acetate, pH 4.5), 10 µL of BACE1 (1.0 U/mL), 10 µL of the substrate (750 nM Rh-EVNLDAEFK-Quencher in 50 mM, ammonium bicarbonate), and 10 µL of compounds (final conc., 100 µM) dissolved in 10% DMSO were incubated for 60 min at 25 °C in the dark. The proteolysis of two fluorophores (Rh-EVNLDAEFK-Quencher) by BACE1 was monitored by the formation of fluorescent donor Rh-EVNL (530–545 nm, excitation; 570–590 nm, emission), the abundance of which was determined by measuring increase in fluorescence excited at 545 nm and recorded at 585 nm. Fluorescence was measured with a microplate spectrophotometer (Molecular Devices). The percent of inhibition (%) was obtained by the following equation: % Inhibition = [1 − (S_60_ − S_0_)/(C_60_ − C_0_)] × 100, where C_60_ was the fluorescence of the control (enzyme, buffer, and substrate) after incubation for 60 min, C_0_ was the initial fluorescence of the control, S_60_ was the fluorescence of the tested samples (enzyme, sample solution, and substrate) after incubation for 60 min, and S_0_ was the initial fluorescence of the tested samples. To account for the quenching effect of samples, the sample solution was added to a separate reaction mixture C, and any reduction in fluorescence by the sample was investigated. The BACE1 inhibitory activity of compounds was expressed in terms of the IC_50_ value (μM required to inhibit proteolysis of the substrate, BACE1, by 50%), as calculated from the log-dose inhibition curve. Quercetin was used as a positive control.

### 4.7. Kinetic Parameters of ***3*** towards AChE and ***4*** towards BACE1 Inhibition

To examine the kinetics of rubrofusarin 6-*O-*β-d-gentiobioside/AChE and nor-rubrofusarin 6-*O-*β-d-glucoside/BACE1 interaction, we employed two complementary kinetic methods, namely Lineweaver–Burk and Dixon plots [54,55]. Specifically, Dixon plots for AChE and BACE1 inhibition by **3** and **4**, respectively, were achieved in the presence of different substrate concentration (0 to 0.6 mM for AChE, and 0 to 750 nM for BACE1). Inhibition constants (*K_i_*) were determined by the interpretation of Dixon plots, where the *x*-axis intercept was taken as *K_i_*. The enzyme assays were performed using previously mentioned methods.

### 4.8. AChE and BACE1 Molecular Docking Simulations

In our study, **1**–**3** were tested for AChE, whereas **2** and **4** into the binding site for BACE1 using the Autodock 4.2 software. The crystal structures of the AChE and BACE1 protein targets were acquired from the RCSB Protein Data Bank with the respective accession codes 1acj and 2wjo, respectively. The co-crystallized ligands, tacrine and QUD, were used to generate the grid box for catalytic inhibition mode, whereas reported allosteric inhibitors, donepezil and PMF, were used to generate the grid box for allosteric inhibition mode [28,30], and their three-dimensional (3D) structures were downloaded from PubChem Compound (NCBI), with compound CIDs of 3152 and 97,332, respectively. The 3D structures of rubrofusarin and its derivatives were drawn with ChemDraw Ultra 12.0 (CambridgeSoft, Cambridge, MA, USA), and their pKa values were computed at crystallographic pH (pH = 7.5) using the MarvinSketch (ChemAxon, Budapest, Hungary). PyMOL 1.7.4 and Ligplot^+^ were used for visualization and analysis of results.

### 4.9. Statistical Analysis 

One-way ANOVA and Student’s *t* test (Systat Inc., Evanston, IL, USA) were used for the analysis of statistical significance. All of the results are expressed as the mean ± standard deviation (*n* = 3) of triplicate samples, and were repeated at three individual days.

## 5. Conclusions

The present study showed that **3** and **4** inhibited AChE and BACE1 more efficiently than the other examined derivatives. Our results show the presence of two glucose molecules, arrangement of pyrone at the γ-position of the naphthalene ring, and the substitution of methoxyl group, with hydroxyl at C-8 on the naphthopyrone significantly enhancing AChE inhibitory activity, whereas the presence of a single glucose, a pyrone ring at the γ-position of the naphthalene, and the presence of hydroxyl group at C-8 of the naphthopyrone ring are essential for BACE1 inhibitory activity. Hence, we have successfully identified AChE and BACE1 inhibitors, **3** and **4**, which shows in vitro activity in micromolar range by means of combined ligand- and structure based virtual screening approach. Moreover, we suggest similar derivatives be synthesized and in vivo experiments be conducted to investigate the SARs in depth.

## Figures and Tables

**Table 1:** Electric eel acetylcholinesterase, horse serum butyrylcholinesterase, and human recombinant BACE1 inhibitory activity of compounds isolated from *Cassia obtusifolia.*

Compounds	AChE	BChE	BACE1	*K_i_* Value	Inhibition Type
	IC_50_ (µM) ^a^				
**1**	124.13 ± 1.39	>200	90.01 ± 2.38	NT	NT
**2**	148.08 ± 2.09	>200	190.63 ± 4.68	NT	NT
**3**	15.94 ± 0.32	141.15 ± 1.23	85. 66 ± 3.98	12.83 ^b^	Mixed type ^c^
**4**	86.05 ± 2.01	>200	14.41 ± 2.87	10.01 ^d^	Mixed type ^e^
**5**	83.52 ± 1.56	>200	>200	NT	NT
**6**	82.31 ± 1.63	>200	>200	NT	NT
Berberine ^g^	0.68 ± 0.01	25.77 ± 0.26	NT	NT	NT
Tacrine ^g^	0.25 ^h^	0.01 ^h^	NT	NT	Mixed type ^f^
Donepezil ^g^	0.005 ^h^	1.74 ^h^	NT	NT	NT
Quercetin^g^	NT	NT	21.42 ± 1.04	NT	NT
PMF ^g,i^	NT	NT	59.8 ^h^	NT	NT

^a^ The 50% inhibitory concentration (IC_50_) values (μM) were calculated from a log dose inhibition curve and expressed as mean ± S.E.M of triplicate experiments. ^b,d^ AChE and BACE1 inhibition constants (*K_i_*), respectively, were determined using a Dixon plot. ^c,e^ AChE and BACE1 inhibition type, respectively, were determined using a Lineweaver-Burk plot. ^f^ BChE inhibition type obtained from the literature [26]. ^g^ Positive controls. ^h^ IC_50_ values obtained from the previously reported literatures (tacrine for AChE and BChE [27]; donepezil for AChE [28]; donepezil for BChE [29]; PMF for BACE1 [30]). ^i^ 3,5,7,3′,4′-pentamethoxyflavone. *NT* Not tested.

**Table 2:** Docking affinity scores and possible H-bond formation to AChE (1acj) active sites by **1**, **2**, and **3** along with reported inhibitors.

Compounds	Binding Energy (Kcal/mol) ^a^	No. of H-Bonds	H-Bonds Interacting Residues	Van Der Waals Interacting Residues
**1** (Allosteric inhibition mode)	−7.95	3	Gln69, Tyr70	Asp72, Trp84, Asn85, Pro86, Gly117, Gly118, Tyr121, Ser122, Gly123, Leu127, Tyr130
**2** (Allosteric inhibition mode)	−7.51	5	Tyr121, Arg289, Tyr334, Phe288	Tyr70, Asp72, Trp279, Ser286, Ile287, Phe290, Phe330, Phe331
**3** (Mixed inhibition mode)	−9.06	7	Tyr70, Asn85, Ser122, Glu199, His440	Val71, Asp72, Gln74, Trp84, Gly117, Gly118, Tyr121, Ser200, Phe290, Phe330, Phe331, Tyr334, Gly441
**3** (Allosteric inhibition mode)	−9.57	3	Tyr70, Asn85, Tyr121	Val71, Asp72, Gly80, Ser81, Trp84, Gly118, Gly119, Ser122, Ser200, Trp279, Ile287, Phe330, Phe331, Tyr334, Gly335, Trp432, Ile439, His440, Tyr442
Tacrine ^b^ (Catalytic inhibitor)	−9.8 ^c^	1	His440	Tyr442, Phe330, Trp84, Trp432, Gly441, Glu199, Ile439
Donepezil ^b^ (Allosteric inhibitor)	−10.6	-	-	Tyr70, Ile275, Asp276, Trp279, Ile287, Phe288, Arg289, Tyr334, Tyr121, Ser286, Phe290, Phe330, Phe331

^a^ Estimated the binding free energy of the ligand receptor complex. ^b^ Positive ligands. ^c^ Reference root mean square deviations (RMSD) value: 0.64.

**Table 3:** Docking affinity scores and possible H-bond formation to BACE1 (2wjo) active sites by **4** and **2** along with reported inhibitors.

Compounds	Binding Energy (Kcal/mol) ^a^	No. Of H-Bonds	H-Bonds Interacting Residues	Van Der Waals Interacting Residues
**4** (Catalytic inhibition mode)	−6.61	6	Asp32, Trp76, Asn37, Ile126, Tyr198	Gly230, Gly34, Val69, Phe108, Asp106, Lys75, Pro70, Tyr71, Ser35, Arg128
**4** (Allosteric inhibition mode)	−8.34	6	Gln303, Gln304, Glu339, Gly156	Ser10, Ala335, Val170, Tyr14, Gly13, Gly11, Thr232, Arg307, Val336, Val361, Ala157, Pro308
**2** (Catalytic inhibition mode)	−5.38	4	Trp76, Lys107, Phe108	Asp32, Gly34, Ser35, Asn37, Val69, Ile110, Trp115, Ile118, Arg128, Asp228, Gly230, Thr231
QUD ^b^ (Catalytic inhibitor)	−11.19^c^	4	Asp228, Asp32, Gly230	Lys107, Lys75, Gly76, Leu30, Thr231, Val69, Tyr198, Ile226, Phe108, Gly34, Arg235, Ser35, Tyr71, Ile118
PMF ^b^ (Allosteric inhibitor)	−6.5	1	Ser10	Gly11, Ala157, Ala168, Val170, Thr232, Gln304, Arg307, Pro308, Tyr320, Ala335, Glu339

^a^ Estimated the binding free energy of the ligand receptor complex. ^b^ Positive ligands. ^c^ RMSD value: 0.73.
